# Intimate Partner Violence and Prenatal Oral Health Experiences

**DOI:** 10.21203/rs.3.rs-3137055/v1

**Published:** 2023-07-17

**Authors:** Alexander Testa, Jacqueline Lee, Dylan B. Jackson, Rahma Mungia, Kyle T. Ganson, Jason M. Nagata

**Affiliations:** The University of Texas Health Science Center at Houston; Boise State University; Johns Hopkins University; The University of Texas Health Science Center at San Antonio; University of Toronto; University of California, San Francisco

**Keywords:** Intimate partner violence, pregnancy, oral health, maternal health, PRAMS

## Abstract

**Background:**

Intimate partner violence (IPV) is a significant public health issue, and when experienced during pregnancy, IPV poses substantial harms for maternal health. Still, limited research has examined how IPV may influence prenatal oral health and dental care utilization. This study aims to investigate the relationship between IPV during pregnancy and women’s oral health experiences.

**Data:**

Data are from the Pregnancy Risk Assessment Monitoring System from 2016 through 2019 (*N* = 85,289). Multivariable logistic regression analyses were used to examine the association between physical IPV during pregnancy and various oral health experiences.

**Findings:**

Women who experienced prenatal physical IPV reported worse oral health experiences during pregnancy, including being more likely to report not knowing it was important to care for their teeth, not talking about dental health with a provider, needing to see a dentist for a problem, going to see a dentist for a problem, as well as having more unmet dental care needs.

**Conclusions:**

Together, these findings indicate that women who experience physical IPV during pregnancy have lower knowledge of prenatal oral health care, more oral health problems, and greater unmet dental care needs. Given the risk associated with IPV and oral health problems for maternal and infant health, the study findings point to greater attention toward the oral health needs of IPV-exposed pregnant women.

## INTRODUCTION

Physical intimate partner violence (IPV)—physical aggression or abuse by a romantic partner—is a traumatic life event that can harm health and well-being.^1^ Physical IPV can be especially detrimental when it is experienced during pregnancy. Estimates suggest that over 300,000 pregnant women experience IPV annually,^2^ and research has connected IPV to adverse health consequences for both maternal and infant health, including physical injury, preterm birth, and maternal and neonatal death.^3–6^ Even so, oral health is one key but overlooked area that IPV may harm.

Oral health is strongly connected to general health and quality of life,^7^ and poor oral health is associated with other systemic diseases due to the shared link with inflammation.^8^ During pregnancy, women are at heightened risk of oral health problems as changes in diet and hormonal fluctuations can contribute to an increased risk of gingivitis and periodontal disease.^9–11^ While the mechanisms are not fully understood, oral health problems during pregnancy have been found to negatively impact birth outcomes, including preterm birth and low birth weight.^12–14^ Indeed, scholars have noted that “maternal oral disease during pregnancy is a significant public health issue due to its prevalence and life course connections with adverse pregnancy/birth outcomes, early childhood caries, and chronic diseases.”^15^ Due to this growing evidence, recent public health efforts have focused on increasing awareness of the importance of oral health care during pregnancy and improving access to preventative and problem-related dental care for pregnant women.^15,16^ However, women who experience physical IPV may face challenges accessing health care services and be at risk for oral health problems. Even though research demonstrates IPV is associated with physical health problems^1^ and access to health care services in general,^17^ there is limited prior research on the influence of IPV on the oral health experiences of women, especially during pregnancy. For instance, one study found that women who experienced IPV during the prenatal period had elevated rates of oral health problems during pregnancy, including problems that stem from physical violence, including painful gingivae, toothache, and an injury to the mouth, teeth, or gingivae.^18^ However, there is a lack of research on a broad range of oral health experiences that might be influenced by IPV including general oral health problems, oral health literacy, dental care utilization, and unmet dental care needs, despite reasons to suspect that IPV might serve as a risk factor that undermines oral health and oral health care utilization during pregnancy.

First, studies of pregnant women who experience abuse often report that their partners attempt to isolate them from support systems.^19–21^ In such cases, an abusive partner may actively prevent a woman from accessing health care services, including dental care.^17^ Moreover, women may not want to deal with the trouble of this process, particularly if they feel that preventative dental care is not that critical, given the host of other issues they are dealing with. Second, and relatedly an abusive partner may interfere with health care visits or treatment to prevent detection of the abuse by a medical provider.^22^ For instance, one recent study found that women who experienced IPV during pregnancy were less likely to receive adequate prenatal care and faced greater barriers to prenatal care services.^23^ However, no research has assessed the relationship between IPV and patterns of dental care utilization. Third, oral health care is often costly, and dental care requires a higher proportion of out-of-pocket spending than other medical expenses.^24,25^ A physically abusive partner may exercise economic dependence that may discourage pregnant women from pursuing oral health care and impact the quality of dental care when it is received.^19,21,26^

In turn, if women experiencing IPV are not receiving regular preventive care, this can lead to more oral health problems and less knowledge about the importance of proper oral health care during pregnancy. In addition, aside from oral health problems that may emerge from a lack of preventive care, IPV can increase oral health problems through traumatic injury from physical violence.^27–29^ Finally, if women who experience IPV during pregnancy have more oral health care needs, but IPV also reduces the likelihood of receiving dental care services, then it is also likely women who experience IPV will be less likely to receive oral health treatment for a problem when they need it.

Given the lack of research on IPV and oral health during pregnancy, the current study draws on state surveillance system data of recent mothers to investigate the association between prenatal physical IPV and a range of oral health experiences during pregnancy.

## METHODS

### Data

The data for this study is from the Pregnancy Risk Assessment Monitoring System (PRAMS). The PRAMS is a population-based surveillance system of live births conducted annually by the Centers for Disease Control and Prevention and state health departments. Each participating site (i.e., states and territories) uses birth certificate records to collect a stratified systematic sample of approximately 100 to 250 mothers who delivered a live birth. The PRAMS data are derived from three primary sources: (1) birth certificates, (2) state and territory vital record systems, and (3) survey responses via a questionnaire. Surveys are distributed to mothers in a series of three mailings made 2–4 months after birth. Nonresponders are followed up with a series of 15 phone calls made throughout a 2-to-3-week period following the last mailing attempt. Sites are included in PRAMS if a minimum response rate has been met, set at 55% in 2015–2017 and 50% since 2018.

The PRAMS survey comprises different questionnaire types that determine the final sample in the current study. A core survey is distributed to all participating sites, which includes questions about the following topics: attitudes about pregnancy, preconception care, prenatal care, Medicaid and WIC participation, breastfeeding, cigarette and alcohol use, health insurance coverage, physical abuse, infant health care, and contraception use. A subset of sites also administered questions from a pretested list developed by the CDC or individual states. This latter questionnaire includes questions about oral health experiences. Therefore, the current study uses data from the 31 states from 2016–2019 with available data on oral health experiences and the other variables used in the analysis (*N* = 85,289 recent mothers). The full list of states and years included in the study are reported in Appendix A. Additional information about the PRAMS survey and methodology is available in Shulman et al.^30^ The use of the PRAMS data for this study was approved by the CDC institutinal review board as part of the external reseracher data sharing agreement.

### Independent Variable

*Physical Intimate Partner Violence* is measured using survey questions asking respondents whether they experienced physical abuse during their pregnancy. Specifically, women were asked two questions: (1) “During your most recent pregnancy, did your husband or partner push, hit, slap, kick, choke, or physically hurt you in any other way?” and (2) “During your most recent pregnancy, did an ex-husband or ex-partner push, hit, slap, kick, choke, or physically hurt you in any other way?” Using these questions, we created a binary variable where women who responded affirmatively to either of these questions were coded as experiencing physical IPV (1) and those who answered no to both were coded as not experiencing physical IPV (0).

### Dependent Variables

The six dependent variables come from a series of questions regarding the mother’s self-reported oral health experiences during pregnancy. Consistent with prior research, all are coded in the direction of risk.^31,32^ *Didn’t Know Importance of Oral Health Care* is measured using a question asking “Did you know it was important to care for your teeth and gums during your pregnancy?” (1 = no, 0 = yes). *Didn’t Talk about Dental Health with Provider* is measured from a survey question asking: “Did a dental or other health care worker talk with you about how to care for your teeth and gums?” (1 = no, 0 = yes). *No Dental Prophylaxis* is based on a question asking, “During your most recent pregnancy, did you have your teeth cleaned by a dentist or dental hygienist?” (1 = no, 0 = yes). *Needed to see Dentist for a Problem* is measured from an item asking, “Did you need to see a dentist for a problem?” (1 = yes, 0 = no). *Visited Dentist for a Problem* is measured from a question asking, “Did you go to see a dentist or dental clinic about a problem?” (1 = yes, 0 = no). Finally, *Visited a Dentist, Conditional on Needing to See a Dentist* is a binary variable based on a subsample of respondents who responded affirmatively to the question inquiring about needing to see a dentist for a problem but who also replied “no” when asked if they visited a dentist for a problem.

### Control Variables

Control variables are included to account for demographic and socioeconomic characteristics of the mother, including *mother’s age* (< 24, 25–29, 30–34, and 35 or older), *mother’s race/ethnicity* (White, Hispanic, Black, Native American, Asian or Other), *currently married* (1 = currently married; 0 = not currently married), *educational attainment* (less than high school, high school graduate, some college, college graduate), *household income* (≤$16,000, $16,0001-$40,000, $40,001-$85,000, >$85,000), and *number of financial dependents* (range 0–7), pre-pregnancy *body mass index* (underweight, normal weight, overweight, obese), *number of prior births* (0, 1, 2, 3 or more), and whether a mother reported having *no dental insurance*, which is measured using a question asking, “Did you have insurance to cover dental care during your pregnancy” (1 = no, 0 = yes). Finally, control variables are also included for the state of residence and year of birth.

### Statistical Analysis

The analysis is performed in three stages. First, the descriptive statistics for the analytic sample are presented. Next, the bivariate association between oral health experiences and IPV is examined using a two-tailed t-test. Third, multiple logistic regression analyses adjusting for control variables are performed. All analyses were adjusted for survey weights and strata information using the SVY command in Stata Version 17.

## RESULTS

Table 1 presents the descriptive statistics for the analytic sample (*N* = 85,289). Overall, 1.8% of respondents reported physical IPV during their most recent pregnancy. Across oral health experience measures, 11.9% of the sample reported not knowing it was important to care for their teeth, 47.5% did not talk about dental health with a provider, 51.5% did not have dental prophylaxis, 18.2% reported needing to see a dentist for a problem, 13.8% reported having visited a dentist for a problem, and among those who reported needing to see a dentist for a problem (*N* = 16,100), 68.3% reported having visited a dentist for a problem.

**Figure 1:**
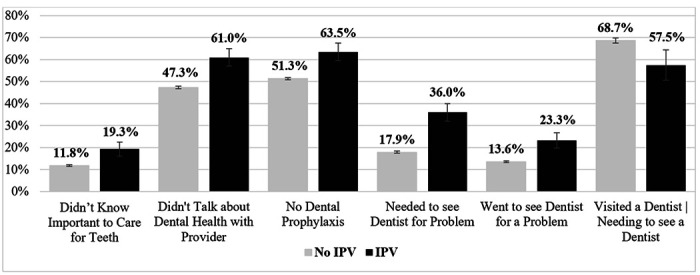
Dental Health Measures by Intimate Partner Violence *Note:* two-sample test for proportions between no IPV and IPV are statistically significant at the α = .01 level for all variables above.

Next, to assess how these patterns vary by physical IPV exposure during pregnancy, Fig. 1 displays the dependent variables stratified by IPV status. The results of two-tailed t-tests demonstrate that women who experienced physical IPV during pregnancy reported worse oral health experiences across all measures. Specifically, IPV exposure was associated with reporting not knowing it was important to care for teeth during pregnancy (19.3% vs. 11.8%, *p* < .001), not talking about dental health with a provider (61.0% vs. 47.3%, *p* < .001), no dental prophylaxis (63.5% vs. 51.3%, *p* < .001), needing to see a dentist for a problem (36.0% vs. 17.9%, *p* < .001), having gone to see a dentist for a problem (23.3% vs. 13.6%, *p* < .001), and having unmet dental care needs (68.7% vs. 57.5%, *p* < .01).

Table 2 presents the oral health measures regressed on IPV adjusting for covariates. Findings show that women who experienced IPV during pregnancy were significantly more likely to report not knowing the importance of oral health care (Odds Ratio [OR] = 1.403, 95% Confidence Interval [CI] = 1.138, 1.729), not having an oral health discussion with their provider (OR = 1.451, 95% CI = 1.217, 1.729), needing to see a dentist for a problem (OR = 1.731, 95% CI = 1.445, 2.072) and having visited a dental care provider for a problem (OR = 1.429, 95% CI = 1.164, 1.756). Finally, restricting to the subsample of women who reported needing to see a dentist for a problem (*n* = 16,100), the results in Model 6 show that IPV exposure had a marginally significant but negative, association with visiting a dentist for a problem. Overall, IPV exposed women were about 25% less likely to see a dentist for a problem conditional on needing to see a dentist for a problem (OR = .746, CI = .555, 1.004), indicating that IPV-exposed women have more unmet dental care needs.

## DISCUSSION

This study aimed to examine the relationship between a mother’s experiences with physical IPV during pregnancy and a variety of oral health experiences. The findings revealed four general patterns about the relationship between IPV and oral health. First, IPV was associated with greater odds of not knowing it was important to care for oral health during pregnancy and not talking with an oral health provider about oral health during pregnancy, which indicates that IPV is related to lower oral health knowledge during pregnancy. Second, women who experienced IPV during pregnancy were more likely to report needing to see a dentist for a problem and going to see a dentist for a problem indicating that IPV is associated with more oral health problems. Third, IPV was related to being less likely to see an oral health care provider for a problem, conditional on needing oral health care for a problem, highlighting that IPV is related to more unmet oral health care needs. Finally, while the results showed that in a bivariate model, IPV was related to a lower likelihood of receiving a dental cleaning during pregnancy, the results of the multiple regression analysis adjusting for control variables found no statistical difference in the odds of receiving dental prophylaxis during pregnancy among women with and without IPV exposure.

### Limitations and Future Directions

Before discussing the implications of these results, it is important to highlight some limitations that can be expanded upon in future research. First, the measure of IPV used in this study refers to physical violence, and therefore the study findings are not necessarily generalizable to other forms of IPV including emotional, psychological, or sexual abuse. Second, because the measure of IPV is focused on the violence that occurs in the contexts during pregnancy, the results cannot speak to the influence of IPV at earlier points in life on oral health experiences during pregnancy. Third, the binary nature of the independent variable captures the presence of whether IPV occurred. However, we lack details on the frequency, duration, and severity of IPV and when the IPV occurred during pregnancy. Fourth, the questions about oral health experiences during pregnancy lack information on specific types of oral health problems such as dental caries, periodontitis, or traumatic dental injuries. Future research that examines the relationship between IPV and more detailed measures of dental health issues would be valuable. Fifth, the IPV and oral health experiences measures are self-reported and can be subject to recall bias or social desirability bias. Finally, because the PRAMS is a cross-sectional survey, this study cannot establish a causal association between IPV and oral health experiences during pregnancy.

### Public Health Implications

Considering these limitations, the results hold important implications for improving oral health experiences among women experiencing IPV. The general pattern detected in this study is that IPV exposure is associated with (1) less knowledge about oral health during pregnancy, (2) greater oral health care needs, and (3) more unmet dental care needs. Therefore, these findings highlight the need for better oral healthcare access among women who have experienced IPV. One way to expand access to dental care knowledge is by providing information on proper oral health during pregnancy as a component of prenatal care. Such information can include information about standard oral health guidelines during pregnancy and locations of local affordable dental care providers. Second, expanding oral health treatment to domestic violence shelters can be a useful way to reach IPV-exposed women who are out of the reach of the traditional medical care system.^33^ Finally, another important implication of this study is increasing dental care providers’ knowledge about IPV. Because many IPV-related injuries are to the head, neck, and face, dental care providers play an important role in detecting IPV and providing resources to aid IPV victims. Even so, studies show that a sizeable portion of dental providers do not feel they have sufficient training to assist victims of IPV or to screen for IPV properly.^34,35^ Thus, it would be beneficial to expand educational offerings on IPV in dental schools and continuing education programs.^35^ Indeed, evidence suggests domestic violence education can improve the dental care provided to survivors of domestic violence.^33^

## CONCLUSIONS

The current study’s findings showed that women who experienced physical IPV during pregnancy also exhibited more oral health problems, lower oral health knowledge, and greater unmet oral health care needs. Considering the profound risks that both IPV and oral health problems can pose for maternal and child health, the findings point to the need for efforts to reduce the prevalence of IPV during pregnancy and expand oral health care services to IPV-exposed populations.

## Figures and Tables

**Table 1:** Summary Statistics from Pregnancy Risk Assessment Monitoring System, 2016-2019 (*N* = 85,289)

Variables	%/Mean (SD)
*Oral Health Experiences*	
Didn’t Know Important to Care for Teeth	11.9%
Didn’t Talk about Dental Health with Provider	47.5%
No Dental Prophylaxis	51.5%
Needed to see Dentist for a Problem	18.2%
Visited Dentist for Problem	13.8%
Visited a Dentist | Needing to see a Dentist	68.3%
Intimate Partner Violence	1.8%
*Maternal Age*	
<24	20.3%
25-29	29.1%
30-34	31.0%
35+	19.6%
*Maternal Race/Ethnicity*	
White	63.0%
Hispanic	15.0%
Black	13.3%
Other Race/Ethnicity	8.6%
*Maternal Educational Attainment*	
Less than High School	9.2%
High School Graduate	22.8%
Some College	27.1%
College Graduate	40.9%
Married	65.3%
*Number of Prior Births*	
0	39.1%
1	33.7%
2	16.1%
3+	11.1%
*Body Mass Index*	
Underweight	3.1%
Normal Weight	44.1%
Overweight	24.8%
Obese	28.0%
*Household Income*	
≤ $16,000	17.4%
$16,000, $40,000	22.6%
$40,001 – $85,000	30.1%
> $85,000	29.9%
Number of Dependents	2.94 (1.37)
No Dental Insurance	19.7%

*Abbreviations:* SD = Standard deviation

*Note:* Visited a Dentist | Needing to see a Dentist available for 16,100 respondents who reported needing to see a dentist for a problem

**Table 2:** Logistic Regression of Oral Health Experiences During Pregnancy on Intimate Partner Violence and Other Covariates

	Model 1:		Model 2:		Model 3:		Model 4:		Model 5:		Model 6:	
	Didn’t Know Important to Care for Teeth		Didn’t Talk about Dental Health		No Dental Prophylaxis		Needed to see Dentist for a Problem		Went to see Dentist for a Problem		Visited a Dentist | Needing to see a Dentist	

Variables	OR	95% CI	OR	95% CI	OR	95% CI	OR	95% CI	OR	95% CI	OR	95% CI
Intimate Partner Violence	1.403**	(1.138, 1.729)	1.451***	(1.217, 1.729)	1.012	(0.849, 1.207)	1.731***	(1.445, 2.072)	1.429***	(1.164, 1.756)	0.746†	(0.555, 1.004)

Control Variables	✓		✓		✓		✓		✓		✓	

**Observations**	**85,289**		**85,289**		**85,289**		**85,289**		**85,289**		**16,100**	

*** *p*<0.001

** *p*<0.01

* *p* <0.05

† *p* <.10

*Note:* Control variables include maternal age, maternal race/ethnicity, maternal educational attainment, marital status, number of prior births, prepregnancy body mass index, household income, number of financial dependents, does not have dental insurance, state of residence, and year of birth

## Data Availability

The datasets generated and/or analyzed during the current study are not publicly available due to the nature of Pregnancy Risk Assessment Monitoring System not being publicly available. Data used in this study can be requested at https://www.cdc.gov/prams/index.htm. Queries about the data can be directed to Alexander Testa: alexander.testa@uth.tmc.edu
